# SARS-CoV-2 molecular epidemiology in Slovenia, January to September 2021

**DOI:** 10.2807/1560-7917.ES.2023.28.8.2200451

**Published:** 2023-02-23

**Authors:** Sandra Janezic, Aleksander Mahnic, Urška Kuhar, Jernej Kovač, Barbara Jenko Bizjan, Tom Koritnik, Tine Tesovnik, Robert Šket, Uroš Krapež, Brigita Slavec, Tadej Malovrh, Tadej Battelino, Maja Rupnik, Tjasa Zohar Cretnik, NLZOH COVID-19 team, CISLD NGS team, Members of the NLZOH COVID-19 team, Mateja Borinc, Martin Bosilj, Mojca Cimerman, David Cvetko, Tina Cvetković, Nika Gobec, Andrej Golle, Tatjana Harlander, Maša Jarčič, Daša Kavka, Monika Korošec, Marjana Petrevčič, Mitja Rak, Mateja Ravnik, Matjaž Retelj, Gašper Strugar, Alenka Štorman, Kaja Tominc, Nika Volmajer, Members of the CISLD NGS team, UMC Ljubljana, Katarina Kozmos, Ana Grom, Maruša Debeljak, Marko Pokorn

**Affiliations:** 1 National Laboratory for Health, Environment and Food, Maribor, Slovenia; 2 Institute of Microbiology and Parasitology, Veterinary Faculty, University of Ljubljana, Ljubljana, Slovenia; 3 Clinical Institute of Special Laboratory Diagnostics, University Children's Hospital, University Medical Centre Ljubljana, Ljubljana, Slovenia; 4 Institute for Poultry, Birds, Small Mammals, and Reptiles, Veterinary Faculty, University of Ljubljana, Ljubljana, Slovenia; 5 Members of the NLZOH COVID-19 team are listed under Collaborators; 6 Members of the CISLD NGS team, UMC Ljubljana are listed under Collaborators

**Keywords:** SARS-CoV-2 genomic surveillance, B.1.258.17 variant, Alpha variant, Delta variant, Slovenia, Virus growth kinetics

## Abstract

**Background:**

Sequencing of SARS-CoV-2 PCR-positive samples was introduced in Slovenia in January 2021. Our surveillance programme comprised three complementary schemes: (A) non-targeted sequencing of at least 10% of samples, (B) sequencing of samples positive after PCR screening for variants of concern (VOC) and (C) sequencing as per epidemiological indication.

**Aim:**

We present the analysis of cumulative data of the non-targeted surveillance of SARS-CoV-2 and variant-dependent growth kinetics for the five most common variants in Slovenia for the first 9 months of 2021.

**Methods:**

SARS-CoV-2 PCR-positive samples, from January to September 2021, were selected for sequencing according to the national surveillance plan. Growth kinetics studies were done on Vero E6 cells.

**Results:**

Altogether 15,175 genomes were sequenced and 64 variants were detected, of which three successively prevailed. Variant B.1.258.17 was detected in ca 80% of samples in January and was replaced, within 9 weeks, by the Alpha variant. The number of cases decreased substantially during the summer of 2021. However, the introduction of the Delta variant caused a fourth wave and completely outcompeted other variants. Other VOC were only detected in small numbers. Infection of Vero E6 cells showed higher replication rates for the variants Alpha and Delta, compared with B.1.258.17, B.1.258, and B.1.1.70, which dominated in Slovenia before the introduction of the Alpha and Delta variants.

**Conclusion:**

Information on SARS-CoV-2 variant diversity provided context to the epidemiological data of PCR-positive cases, contributed to control of the initial spread of known VOC and influenced epidemiological measures.

**Key public health message**

**What did you want to address in this study?**

The aim of this study was to review the national SARS-CoV-2 genomic surveillance programme and to analyse collected data on virus variants that circulated in Slovenia for the first 9 months of 2021. In addition, we compared the growth of the viruses in cultured cells for the three most common variants that circulated in Slovenia at that time.

**What have we learnt from this study?**

Three successive waves have shaped the epidemiology of COVID-19 in Slovenia since January 2021. Each wave was dominated by a single variant (B.1.258.17, Alpha and Delta), while the diversity of other, not frequent variants decreased in each wave. The linage B.1.258.17 is an example of a nationally prevailing variant that has not commonly been detected in other countries. Each successive competing variant also grew faster on cultured cells.

**What are the implications of your findings for public health?**

The national molecular surveillance programme enabled us to monitor SARS-CoV-2 variants circulating in Slovenia and to quickly detect new variants of concern. This information provided the context for risk assessment and contributed to the decisions on appropriate public health measures. It has also been used in epidemiological contact tracing for the first cases infected with newly introduced variants and thereby slowed their spread.

## Introduction

In Slovenia, the first patient with severe acute respiratory syndrome coronavirus 2 (SARS-CoV-2) was detected on 4 March 2020, and the coronavirus disease (COVID-19) epidemic in Slovenia was declared on 12 March 2020. The first epidemic wave in the spring of 2020 was mild compared with other European Union (EU) countries [1]. Between March and June 2020, the maximum number of daily positive PCR tests reached 61, and the maximum number of daily deaths was six for the entire Slovenian population of 2.09 million. In September 2020, the numbers of positive cases started to rise dramatically, from 108 to more than 2,500 cases daily in October 2020. During the second wave, the 7-day average of > 1,000 positive PCR tests per day remained stable for more than 3 months, from 23 October 2020 to 7 February 2021 [1]. By the end of September 2021, more than 290,000 people had been PCR-positive, and 4,942 had died due to COVID-19 in Slovenia (2,365 deaths per million).

Almost all of the SARS-CoV-2 PCR diagnostics in Slovenia were performed by two large laboratories: the National Laboratory for Health, Environment and Food (NLZOH) and the Institute for Microbiology and Immunology (IMI) at the Medical faculty, University of Ljubljana. While IMI mostly, but not exclusively, serves the central Slovenian statistical region and one large university teaching hospital (University Medical Centre Ljubljana), NLZOH laboratories cover different areas in Slovenia.

SARS-CoV-2 variant monitoring was established mainly as a response to the emergence of the Alpha variant (Phylogenetic Assignment of Named Global Outbreak (Pango) lineage designation: B.1.1.7) [2], and NLZOH was assigned to be the national coordinator. The national surveillance plan included whole genome sequencing (WGS) of at least 10% of PCR-positive samples, sequencing of other samples as per the request from epidemiologists (following guidelines from the European Centre for Disease Prevention and Control (ECDC) [3], sequencing samples positive for variants of concern (VOC; as detected by specific RT-PCR), monitoring variants in sewage waters, and characterising selected variants on cell culture models. These activities are performed by five different Slovenian institutions.

The NLZOH and IMI have been sequencing individual PCR-positive samples since April 2020, and five genome sequences for Slovenia were available on GISAID in May 2020, at the time of the first pan-European analysis of circulating SARS-CoV-2 variants [4]. In Slovenia, WGS-based SARS-CoV-2 surveillance was established in January 2021 and was fully operational in February 2021, when weekly numbers of sequenced samples reached at least 10% of PCR-positive samples. By the end of October 2021, Slovenia had uploaded more than 31,000 genomes to the GISAID repository.

Here, we represent the analysis of cumulative data of the non-targeted surveillance of SARS-CoV-2-positive samples from the first 9 months of year 2021 that were diagnosed at NLZOH and sequenced in collaboration with the Clinical Institute of Special Laboratory Diagnostics at the University Children’s Hospital, University Medical Centre Ljubljana. The dataset originates from all Slovenian regions and therefore reflects SARS-CoV-2 epidemiology in Slovenia. In addition, representatives of the five most frequent SARS-CoV-2 variants were isolated and tested for growth kinetics on Vero E6 cells.

## Methods

### Sample selection

The NLZOH is a large public health institution providing, among other activities, also microbiological diagnostics at seven locations that cover all Slovenian statistical regions. From the start of the COVID-19 pandemic, NLZOH laboratories performed approximately half of the SARS-CoV-2 PCR tests in Slovenia. During the study period, PCR tests were performed (i) for all cases with respiratory symptoms and thus suspected to be infected with SARS-CoV-2 and (ii) as a confirmation test for asymptomatic cases with positive results after screening with rapid antigen tests.

The SARS-CoV-2 PCR-positive samples detected at NLZOH were included in WGS through three schemes: (A) non-targeted surveillance with direct inclusion of at least 10% of positive samples, (B) non-targeted surveillance based on PCR screening for variants and (C) targeted surveillance as per epidemiological indication (travellers, reinfections, post-vaccination infections or patients treated in an intensive care unit).

Scheme A was initiated in January 2021 and since the beginning of February 2021, at least 10% of all SARS-CoV-2 PCR-positive samples from all NLZOH diagnostic laboratories have been subjected to WGS. Generally, at each NLZOH location, every 10th PCR-positive sample with a Cq value of < 30 was selected. Occasionally, when a variant of concern or interest was first detected in a particular statistical region, laboratories increased the proportion of sequenced samples from 10% to 30%, to increase the likelihood of detecting this variant. During weeks 24–32 of 2021, when the number of PCR-positive samples at NLZOH decreased to < 500 samples per week, all PCR-positive samples with Cq values of < 30 were subjected to WGS. Each NLZOH diagnostic laboratory submitted the RNA extracts to a dedicated NLZOH location for further processing.

For scheme B, different commercial PCR kits for detecting specific mutations were used based on kit availability and compatibility with available systems and equipment at each NLZOH diagnostic laboratory. Every 10th SARS-CoV-2-positive sample (different from the samples included in surveillance sequencing) was tested for VOC (Alpha, Beta (B.1.351), Gamma (P.1) and Delta (B.1.617.2)). All samples with presumptive VOC, detected by PCR screening, were further subjected to WGS.

### Library preparation, sequencing, and sequence analysis

Illumina-compatible libraries were prepared with target genome amplification with different commercially available kits, depending on their availability. A detailed description of the methodology is provided in the Supplement. Partial library preparation followed by sequencing and bioinformatic analysis were performed at the Clinical Institute of Special Laboratory Diagnostics, University Children's Hospital, University Medical Centre Ljubljana. Part of the samples (n = 1,725 genomes) were sequenced at Eurofins Genomics (Ebersberg, Germany), financially supported by the ECDC.

Consensus genome sequences were obtained by mapping to the reference (for the details see Supplementary Methods). SARS-CoV-2 variants were called with locally installed Pangolin (daily updated version [5]) and mutations were obtained with online Nextclade analysis [6]. All viral genomes used in this study were deposited in the GISAID repository (https://www.gisaid.org).

### Virus isolation and growth kinetics using Vero E6 cells

All work with viruses in cell cultures was performed in a biosafety level 3 (BSL3) laboratory at the Institute of Microbiology and Parasitology, Veterinary Faculty, University of Ljubljana.

The SARS-CoV-2 variants were isolated on Vero E6 cells from samples collected from SARS-CoV-2-positive individuals. Cytopathic effects were observed under an inverted microscope (Eclipse Ts2R, Nikon). The virus titres of the working stocks were determined by titration of the virus isolates on Vero E6 cells and measured as 50% infectious tissue culture dose. Each viral variant was tested in six replicates for each time point, and viral load from the supernatants was measured using the SARS-CoV-2 real-time PCR assay targeting the E gene [7] (a detailed description of the methodology is provided in the Supplement).

## Results

### Genomic surveillance of SARS-CoV-2 in Slovenia

A total of 90,072 samples were SARS-CoV-2 PCR-positive at NLZOH, which represents 56.7% of all PCR-positive samples confirmed in Slovenia from January until the end of September 2021. Of these 90,072 positive samples, 15,175 (16.8%) samples were sequenced. Most sequenced genomes, 13,011 (85.7%), were from non-targeted scheme A, representing 14.5% of SARS-CoV-2-positive samples. An additional 2,164 (14.3%) genomes were sequenced per epidemiological indication (scheme C) or to confirm positive PCR signals for variant detection (scheme B), together representing 2.4% of the PCR-positive samples from NLZOH included here (Table 1). 

**Table 1:** Overview of all sequenced SARS-CoV-2 variants in all NLZOH laboratories, Slovenia, January–September 2021 (n = 15,175)

SARS-CoV-2 variant	Samples scheme A (n = 13,011)		Samples schemes B and C (n = 2,164)		Total (n = 15,175)	
	n	%	n	%	n	%
Delta (B.1.617.2 and AY sublineages)	5,363	41.22	512	23.66	5,875	38.71
B.1.1.7	3,490	26.82	1,410	65.16	4,900	32.29
B.1.258.17	3,567	27.42	169	7.81	3,736	24.62
B.1.258	155	1.19	15	0.69	170	1.12
B.1.1.70	113	0.87	16	0.74	129	0.85
B.1.160	72	0.55	4	0.18	76	0.50
B.1.177	34	0.26	4	0.18	38	0.25
B.1	29	0.22	2	0.09	31	0.20
B.1.1	23	0.18	7	0.32	30	0.20
B.1.525	19	0.15	5	0.23	24	0.16
B.1.149	21	0.16	2	0.09	23	0.15
B.1.221	17	0.13	1	0.05	18	0.12
C.36.3	10	0.08	1	0.05	11	0.07
B.1.139	10	0.08	0	0	10	0.07
B.1.2	9	0.07	0	0	9	0.06
C.36	6	0.05	1	0.05	7	0.05
B.1.351	5	0.04	1	0.05	6	0.04
B.1.526	1	0.01	4	0.18	5	0.03
P.1	2	0.02	2	0.09	4	0.03
B.1.1.318	2	0.02	2	0.09	4	0.03
B.1.1.29	4	0.03	0	0	4	0.03
B.1.146	4	0.03	0	0	4	0.03
B.1.1.159	4	0.03	0	0	4	0.03
B.1.1.58	3	0.02	0	0	3	0.02
B.1.247	3	0.02	0	0	3	0.02
A	2	0.02	1	0.05	3	0.02
B.1.258.4	2	0.02	0	0	2	0.01
B.1.1.317	2	0.02	0	0	2	0.01
B.1.521	2	0.02	0	0	2	0.01
B.1.153	2	0.02	0	0	2	0.01
A.27	2	0.02	0	0	2	0.01
P.1.1	1	0.01	1	0.05	2	0.01
B.1.36	2	0.02	0	0	2	0.01
B.1.236	2	0.02	0	0	2	0.01
B.1.1.294	2	0.02	0	0	2	0.01
B.1.258.7	2	0.02	0	0	2	0.01
B.1.1.47	1	0.01	0	0	1	0.01
B.1.617.1	1	0.01	0	0	1	0.01
B.1.1.222	1	0.01	0	0	1	0.01
B	1	0.01	0	0	1	0.01
B.1.570	1	0.01	0	0	1	0.01
B.1.258.1	1	0.01	0	0	1	0.01
B.55	1	0.01	0	0	1	0.01
B.1.258.12	1	0.01	0	0	1	0.01
B.1.1.130	1	0.01	0	0	1	0.01
B.1.1.228	1	0.01	0	0	1	0.01
B.1.258.18	1	0.01	0	0	1	0.01
B.1.565	1	0.01	0	0	1	0.01
B.1.1.282	1	0.01	0	0	1	0.01
B.1.609	1	0.01	0	0	1	0.01
B.1.621	1	0.01	0	0	1	0.01
B.1.260	1	0.01	0	0	1	0.01
C.16	1	0.01	0	0	1	0.01
B.1.1.39	1	0.01	0	0	1	0.01
B.1.1.64	1	0.01	0	0	1	0.01
A.5	1	0.01	0	0	1	0.01
B.1.1.91	1	0.01	0	0	1	0.01
B.1.367	1	0.01	0	0	1	0.01
B.1.222	1	0.01	0	0	1	0.01
B.1.160.14	1	0.01	0	0	1	0.01
B.1.1.117	0	0	1	0.05	1	0.01
B.1.1.161	0	0	1	0.05	1	0.01
B.1.1.419	0	0	1	0.05	1	0.01
B.1.1.135	0	0	1	0.05	1	0.01

NLZOH: National Laboratory for Health, Environment and Food; SARS-CoV-2: severe acute respiratory syndrome coronavirus 2.

Values are shown separately for scheme A (non-targeted sequencing of 10% of positive samples) and schemes B (confirmation of variants detected with mutation-specific PCR) and C (as per epidemiologist’s indication).

**Figure 1:**
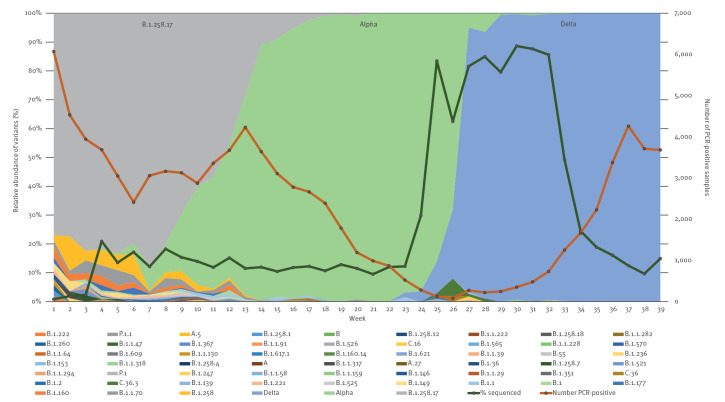
Distribution of SARS-CoV-2 variants, Slovenia, January–September 2021 (n = 15,175)

Altogether, 64 SARS-CoV-2 variants were detected, among which B.1.258.17, Alpha (B.1.1.7) and Delta (B.1.617.2 and AY sublineages) predominated (Table 1, Figure 1). Other variants were detected in much lower numbers or only occasionally. In scheme A, 60 SARS-CoV-2 variants were detected, and an additional four variants, that were not detected in scheme A, were found in the samples from other schemes (Table 1). Further analysis is presented only for scheme A, as it represented variant variability within our sample set well.

SARS-CoV-2: severe acute respiratory syndrome coronavirus 2.

Colours represent the relative abundance of variants with time. Red line: number of PCR-positive samples per week; green line: percentage of sequenced samples.

**Figure 2:**
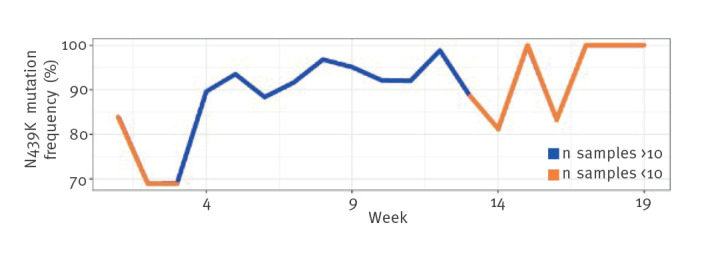
Prevalence of N439K amino acid substitution in the SARS-CoV-2 B.1.258.17 lineage over 19 weeks, Slovenia, January–May 2021 (n = 2,641)

Variant B.1.258.17 was detected in 27.4% of all sequenced samples from 1 January 2021 to 30 September 2021 (Table 2, Figure 1). This variant is characterised by mutations H69del, V70del, L189F, N439K, D614G and V772I (an overview of the substitutions is appended in Supplementary Figure S1). Even though WGS surveillance began when B.1.258.17 was already well established in the Slovenian population, the prevalence of the N439K amino acid (aa) substitution in this variant increased over time (Figure 2).

**Table 2:** Weekly distribution of SARS-CoV-2 variants detected by non-targeted surveillance, Slovenia, January–September 2021 (n = 13,011)

Week	Number of PCR-positive samples	Number of sequenced samples	% sequenced of all PCR-positive samples	Samples with variant B 1.258.17		Samples with variant Alpha		Samples with variant Delta		Samples with other variants		Number of different variants in these samples
				n	%	n	%	n	%	n	%	
1	6,075	52	0.86	40	76.9	0	0	0	0	12	23.1	12
2	4,533	84	1.85	65	77.4	0	0	0	0	19	22.6	7
3	3,944	91	2.31	75	82.4	0	0	0	0	16	17.6	9
4	3,687	772	20.94	630	81.6	3	0.4	0	0	139	18.0	20
5	3,051	413	13.54	344	83.3	2	0.5	0	0	67	16.2	12
6	2,412	414	17.16	330	79.7	16	3.9	0	0	68	16.4	12
7	3,058	370	12.10	325	87.8	30	8.1	0	0	15	4.1	11
8	3,163	577	18.24	463	80.2	55	9.5	0	0	59	10.2	13
9	3,127	480	15.35	334	69.6	95	19.8	0	0	51	10.6	16
10	2,873	400	13.92	241	60.3	136	34.0	0	0	23	5.8	13
11	3,361	399	11.87	226	56.6	155	38.8	0	0	18	4.5	9
12	3,673	556	15.14	251	45.1	259	46.6	0	0	46	8.3	14
13	4,230	487	11.51	138	28.3	338	69.4	0	0	11	2.3	8
14	3,642	435	11.94	47	10.8	386	88.7	0	0	2	0.5	4
15	3,108	325	10.46	29	8.9	290	89.2	0	0	6	1.8	5
16	2,779	328	11.80	17	5.2	307	93.6	0	0	4	1.2	5
17	2,664	326	12.24	8	2.5	311	95.4	0	0	7	2.1	5
18	2,387	255	10.68	2	0.8	252	98.8	1	0.4	0	0	3
19	1,783	229	12.84	1	0.4	228	99.6	0	0	0	0	2
20	1,191	137	11.50	1	0.7	135	98.5	0	0	1	0.7	3
21	989	94	9.50	0	0	94	100.0	0	0	0	0	1
22	868	104	11.98	0	0	104	100.0	0	0	0	0	1
23	523	64	12.24	0	0	62	96.9	1	1.6	1	1.6	3
24	282	84	29.79	0	0	81	96.4	3	3.6	0	0	2
25	121	101	83.47	0	0	87	86.1	11	10.9	3	3.0	5
26	80	50	62.50	0	0	34	68.0	12	24.0	4	8.0	4
27	272	222	81.62	0	0	11	5.0	204	91.9	7	3.2	6
28	219	186	84.93	0	0	12	6.5	172	92.5	2	1.1	3
29	245	195	79.59	0	0	1	0.5	194	99.5	0	0	2
30	352	312	88.64	0	0	1	0.3	309	99.0	2	0.6	4
31	477	418	87.63	0	0	3	0.7	414	99.0	1	0.2	3
32	730	625	85.62	0	0	1	0.2	620	99.2	4	0.6	6
33	1,254	617	49.20	0	0	1	0.2	614	99.5	2	0.3	4
34	1,662	407	24.49	0	0	0	0	407	100.0	0	0	1
35	2,226	420	18.87	0	0	0	0	419	99.8	1	0.2	2
36	3,377	543	16.08	0	0	0	0	543	100.0	0	0	1
37	4,261	532	12.49	0	0	0	0	532	100.0	0	0	1
38	3,711	358	9.65	0	0	0	0	358	100.0	0	0	1
39	3,682	549	14.91	0	0	0	0	549	100.0	0	0	1
**Total**	**90,072**	**13,011**	**14.45**	**3,567**	**27.4**	**3,490**	**26.8**	**5,363**	**41.2**	**591**	**4.5**	**60**

SARS-CoV-2: severe acute respiratory syndrome coronavirus 2.

The surveillance included 10% of all PCR-positive samples (scheme A). The numbers of samples and percentages are specified only for the three predominant variants. For the other variants, only the total numbers of variants are provided.

SARS-CoV-2: severe acute respiratory syndrome coronavirus 2.

Orange lines: weeks where the N439K mutation frequency could be biased because there were fewer than 10 B.1.258.17 samples.

In week 4 of 2021, the Alpha variant emerged and was detected in 0.4% of positive samples. By week 12, the percentage increased to 46.6%, ranging from 25% to 84.4% across different Slovenian statistical regions. The Alpha variant replaced the most prevalent former variant, B.1.258.17, within 10 weeks (Table 2). In week 18 of 2021, the Delta variant was detected for the first time; however, it was found only sporadically until week 25. By week 25, 11 Delta-variant-positive samples were confirmed, which represented 10.9% of sequenced samples. Within only 2 weeks, the Delta variant predominated, representing 91.4% of sequenced samples, and remained the predominant variant (99–100%) until the end of the study period (Table 2, Figure 1).

All three prevalent variants, B.1.258.17, Alpha and Delta, exhibited inter-variant diversity (we append the phylogenetic trees in Supplementary Figure S2, GISAID accession numbers used for construction of these trees are listed in the Supplementary Tables). Regarding the Alpha variant, the distribution of early genomes throughout the phylogenetic tree reflects multiple introductions and sub-lineage evolution. Similar was also observed for B.1.258.17. The phylogenetic analysis of variant B.1.258.17 also included samples collected from October to December 2020 (i.e. before the period focused on by this study). However, the highest inter-variant diversity was observed for the Delta variant. The most frequent aa substitutions in the Delta S gene included T95I, G1167V, S477I, L5F and G181V, while P251L gradually disappeared over time (for mutation frequencies over time please see Supplementary Figure S1).

Other internationally recognised VOC and variants under investigation (VUI) were detected in low numbers, either sporadically or as small-scale transmission events (Table 1). The NLZOH surveillance detected the following variants: Beta (B.1.351; six samples), Gamma (P.1 and P.1.1; six samples), Eta (B.1.525; 24 samples), B.1.1.318 (four samples from the same region but not the same epidemiological cluster), Iota (B.1.526; five samples from the same region), C.36.3 (10 samples from the same statistical region) and B.1.621 (one sample). The variability of other variants was high when B.1.258.17 predominated and much lower when the Alpha (B.1.1.7) and Delta variants (B.1.617.2) predominated (Table 1, Figure 1).

### Variant-dependent growth kinetics in Vero E6 cells

**Figure 3:**
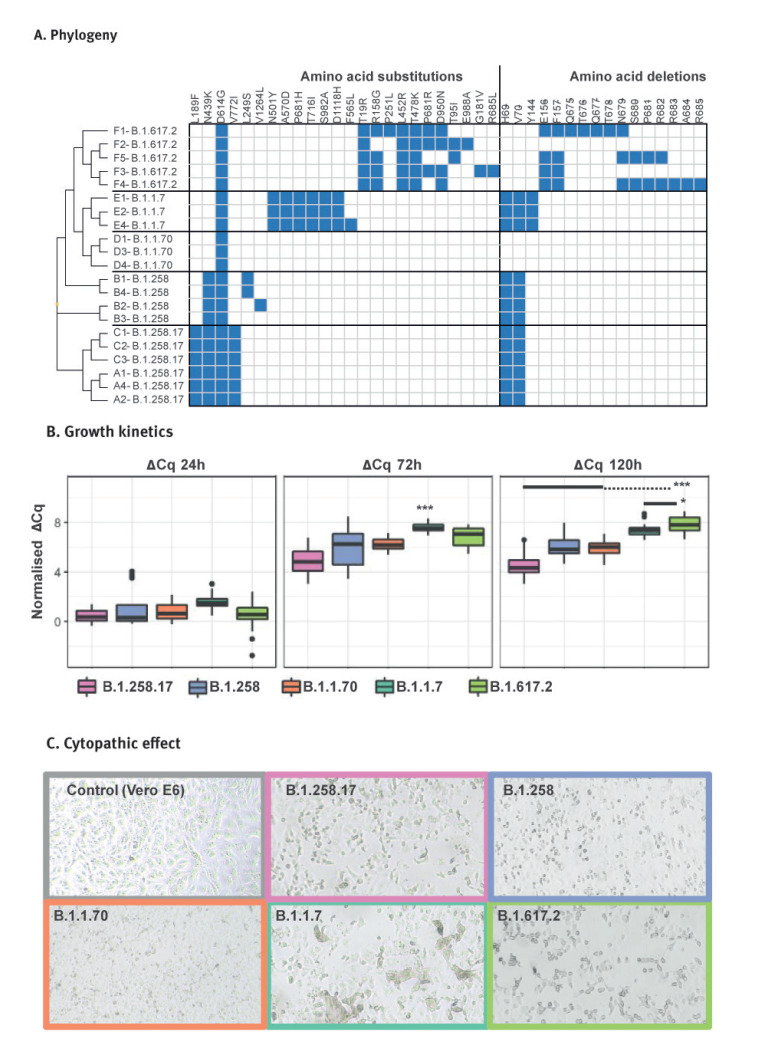
Growth kinetics in Vero E6 cells of the most common SARS-CoV-2 variants, Slovenia, March and July 2021 (n = 21)

CI: confidence interval; Cq: quantification cycle; SARS-CoV-2: severe acute respiratory syndrome coronavirus 2.

The most frequent SARS-CoV-2 variants in Slovenia were additionally tested for growth kinetics in Vero E6 cells. The main goal was to compare the replication rate of B.1.258.17 and other lineages to better understand the biological significance of its early predominance and fast disappearance after the emergence of the Alpha variant. Even though we confirmed inter-variant polymorphisms at aa level (Figure 3A), we focus here on differences according to variant designation (Pango designation v1.2.97), since the effect of individual mutations is beyond the scope of this study.

Panel A: Phylogenetic analysis of variants included in Vero cell line assay. Dendrogram was obtained by full genome alignment with mafft (v7.480) and tree calculation with IQ-TREE (v1.6.1) GTR+G model. We present amino acid substitutions and deletions based on Nextclade analysis (v1.7.4). Panel B: Quantification was performed with the SARS-CoV-2 real-time RT-PCR assay targeting the E gene. All Cq values were normalised against measurements obtained after 2 h of incubation (considered the baseline viral load). All subsequent time points (24, 72 and 120 h) were compared against measurements obtained after 2 h to obtain the difference in virus load (ΔCq). Significance is presented at 95% CI (*) and > 99.9% CI (***). Panel C: Cytopathic effects of different SARS-CoV-2 variants on Vero E6 cells were observed with an inverted microscope (Eclipse Ts2R, Nikon).

After 72 h of incubation, the Alpha variant exhibited the highest rate of replication (p < 0.001), followed by the Delta variant (p < 0.003). All the variants reached the viral load limit after 72 h; however, the Delta variant showed a further increase in viral load after 120 h (p < 0.001). At this time point, the Delta variant replicated at the highest rate (p < 0.001 compared with all variants except B.1.1.7, where p = 0.011) (Figure 3B). Variant B.1.258.17, which predominated in Slovenia in 2020 and early 2021, showed the slowest replication rate among the tested variants, and this was most evident after 120 h of incubation (p < 0.001).

The cytopathic effect on Vero E6 cells was similar between different SARS-CoV-2 variants. Cell death was morphologically manifested as detaching, clumping and rounding of adherent cells. The Alpha variant was an exception for which we observed increased cell–cell fusion and syncytia-like formation (Figure 3C).

## Discussion

The molecular epidemiology of SARS-CoV-2 in Slovenia between January and September of 2021 was characterised by successive waves of three prevailing variants and with an overall decrease in the diversity of circulating variants. Two of the predominant variants, Alpha and Delta, are recognised worldwide as VOC [2,8,9]. The third predominating variant, B.1.258.17, prevailed throughout Slovenia in the beginning of our genomic surveillance (during the first 11 weeks of 2021) but did not show potential to spread globally.

According to the Pango lineage overview page [10], genomes from variant B.1.258.17 were detected mostly in Slovenia (78%), followed by Germany (6%), Switzerland (6%), Sweden (4%) and in Austria (2%), which is the only country among those listed that neighbours Slovenia. Countries with substantial populations that transit through Slovenia (due to holidays or work) have also reported small numbers of B.1.258.17 cases in the same period (Croatia, n = 167; Bosnia and Herzegovina, n = 3; Serbia, n = 1) (information retrieved from GISAID on 8 November 2021). However, the B.1.258.17 sub-lineage diversity was considerably higher in Slovenia, and most sub-lineages that were detected in other European countries had first been reported in Slovenia (see Supplementary Figure S3 for phylogenetic analysis and geographical spread of B.1.258.17 based on data obtained from GISAID and the Supplementary Tables for GISAID accession numbers used for the analysis). This indicates multiple transmission events of B.1.258.17 from Slovenia into other countries (Supplementary Figure S3). We hypothesise that, in addition to strict public health measures, the unsuccessful spread of B.1.258.17 outside Slovenia could also be attributed to low viral fitness of B.1.258.17 compared with variants predominating in other countries at that time.

The reasons for the uncharacteristic spread of this variant in Slovenia remain unclear. Our results with Vero E6 cells showed no advantage of B.1.258.17 compared with B.1.258 or B.1.1.70, variants commonly detected in the period during which B.1.258.17 predominated. We hypothesise that the fixation of the N439K aa substitution, observed in our data, could have contributed to the successful spread of B.1.258.17 in Slovenia (which was later surpassed by the Alpha variant). It has previously been demonstrated that N439K leads to increased affinity for hACE2 cells and evasion of the antibody-mediated immune system [11]. The N439K mutation was confirmed in the B.1.258.17 variant also in other countries.

The Alpha variant was sporadically detected in Slovenia until week 4 of 2021, after which it started to increase exponentially at a national level. The Alpha variant was first detected in Slovenia at the end of December 2020, when it was confirmed in a traveller from the United Kingdom (data not shown). However, more recent sequencing of old samples (as per epidemiologist request due to reinfections) indicated that the Alpha variant was present in Slovenia already in November 2020 (data not shown). The Alpha variant spread and replaced the previously predominant variant B.1.258.17 within ca 10 weeks. During this time, the frequency of other variants decreased compared with the period during which B.1.258.17 predominated. Based on the available data, it is difficult to deduce whether this is solely the consequence of a more virulent and transmissible variant (Alpha) or also a consequence of the epidemiological measures at the time.

In the summer of 2021 (in mid-June), the number of COVID-19 cases in Slovenia decreased to fewer than 50 cases per day. During this period, we observed multiple introductions of the Delta variant. The most prominent was the return of more than 600 students from holidays in Spain, one third of whom were positive for the Delta variant [12]. Many introductions of the Delta variant resulted in fast spread, which quickly replaced the Alpha variant. At the beginning of September 2021, COVID-19 cases in Slovenia increased rapidly, with most of them positive for the Delta variant. 

The observed higher infectivity of the Alpha and Delta variants, as evident from their spread described in the present study and numerous reports worldwide [13-15], is supported by their higher replication rates in Vero E6 cells observed in this study. Vero cells are usually used for the recovery of infectious virus from specimens [16], enrichment of viral particles for downstream analysis [17,18] and viral kinetic studies [19]. In our study, replication rates of the tested variants in Vero E6 cells correlated well with the spread dynamics of the same variants in the Slovenian population. This was most clearly demonstrated by the higher replication rate of the Alpha variant.

## Conclusion

Molecular surveillance at the national level, in particular detection of VOC, provided important information for the appropriate implementation of epidemiological measures. An example of this was the implementation of stricter measures after the detection of the spread of the Alpha variant in Slovenia and introduction of a short lockdown after the rise of Delta. Reports on the variant dynamics were provided weekly to decision-makers and contributed to the context in which data on positive PCR tests, hospitalisation rates, severity and mortality were interpreted and modelled. Another aspect of surveillance was early detection of known VOC (Alpha, Delta). This enabled the implementation of strict epidemic case-based surveillance through contact tracing while the number of cases was still low. This may have led to slower spread and allowed time window, used for vaccination and adjustments to increase hospital and other capacities.

## Supplementary Data

868000 Supplement

## Supplementary Data

95000 Supplementary_TableS1
