# Supplementary material for: SARS-CoV-2 molecular epidemiology in Slovenia, January to September 2021
Source: Euro Surveill. 2023 Feb 23;28(8):2200451. doi: 10.2807/1560-7917.ES.2023.28.8.2200451 (PMC9951254; doi:10.2807/1560-7917.ES.2023.28.8.2200451)
Supplement: Supplement [file 22-00451_JANEZIC_Supplement.pdf]

This supplementary material is hosted by *Eurosurveillance* as supporting information alongside the article »**SARS-CoV-2 molecular epidemiology in Slovenia: January - September 2021"**, on behalf of the authors, who remain responsible for the accuracy and appropriateness of the content. The same standards for ethics, copyright, attributions and permissions as for the article apply. Supplements are not edited by *Eurosurveillance* and the journal is not responsible for the maintenance of any links or email addresses provided therein.

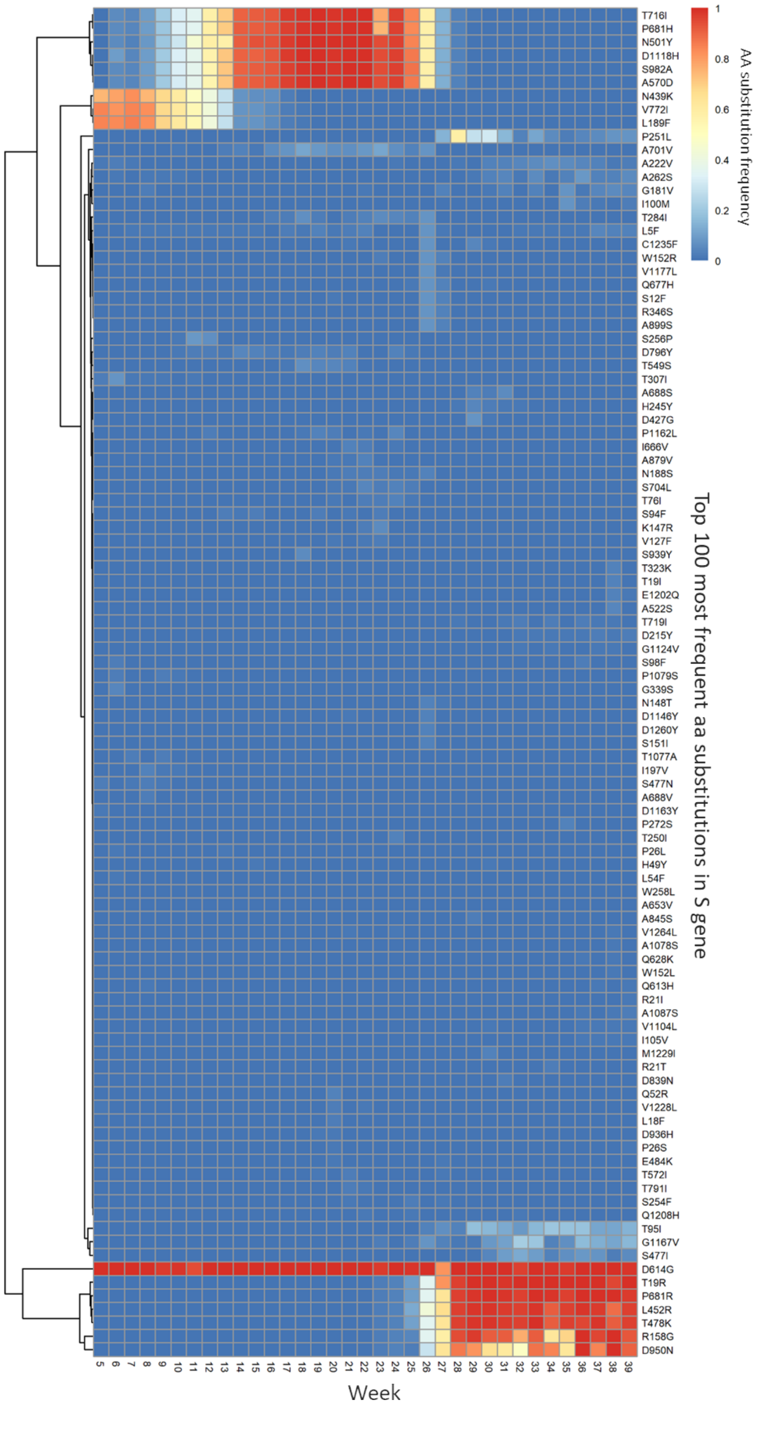

Supplementary Figure S1: Frequency of most common aa substitution in S gene over the period from week 5 to 39 in 2021. AA substitutions were obtained with online Nextclade (v1.7.4). Only genomes with <5% N content were considered for the analysis. The set of variant-specific mutations highlights the successive predominance of B.1.258.17, Alpha and Delta variants.

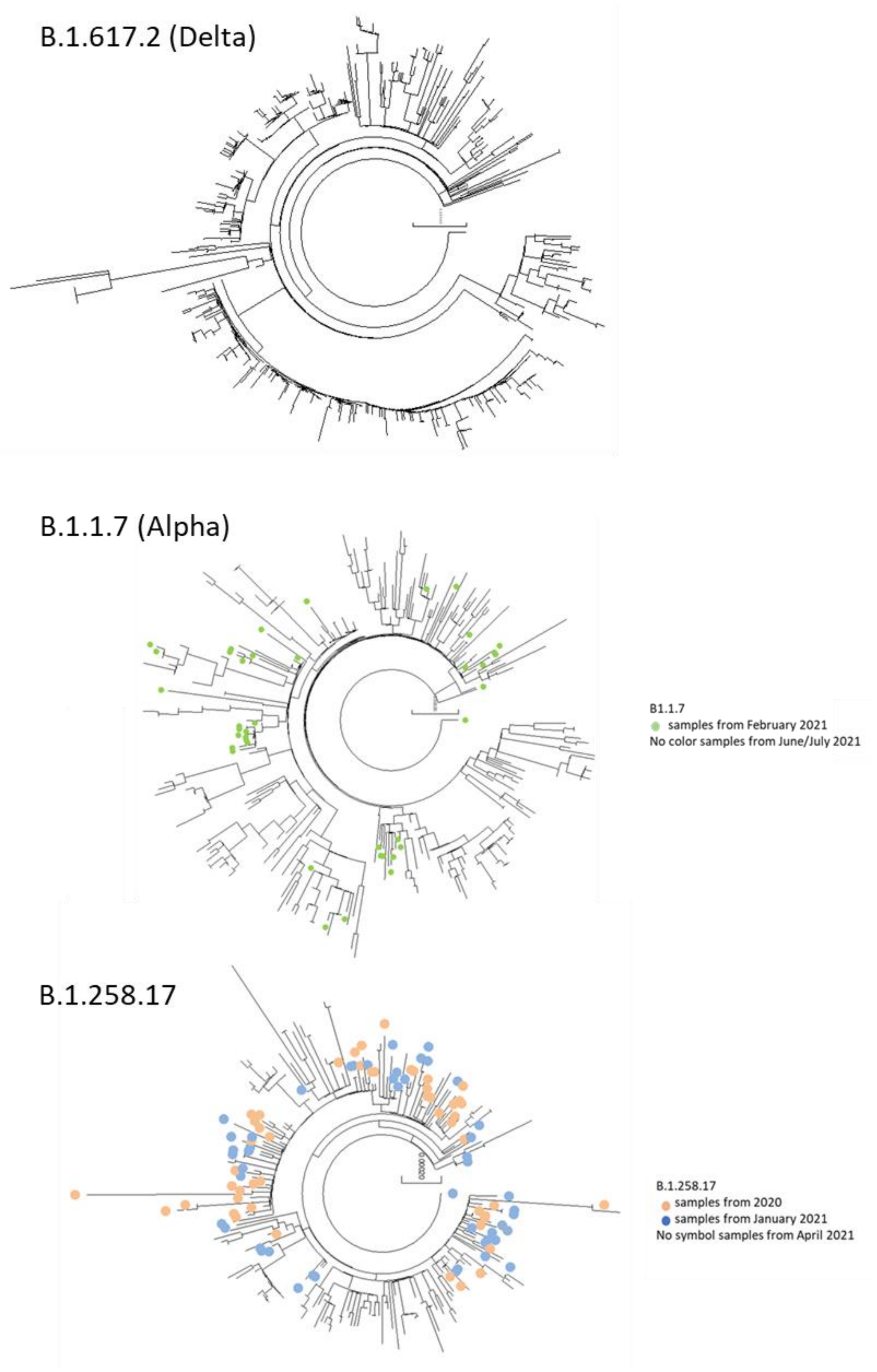

Supplementary Figure S2: Phylogenetic analysis of three most common variants in Slovenia. Dendragram was obtained by full genome alignment with mafft (v7.480) and tree calculation with IQ-TREE (v1.6.1) GTR+G model. On tree presenting B.1.1.7 varinat we highlight early samples from February 2021 (green) in comparison to samples from June/July 2021, while on B.1.258.17 tree we highlight samples from 2020 (orange) and from January 2021 (blue) in comparison to samples from April 2021)

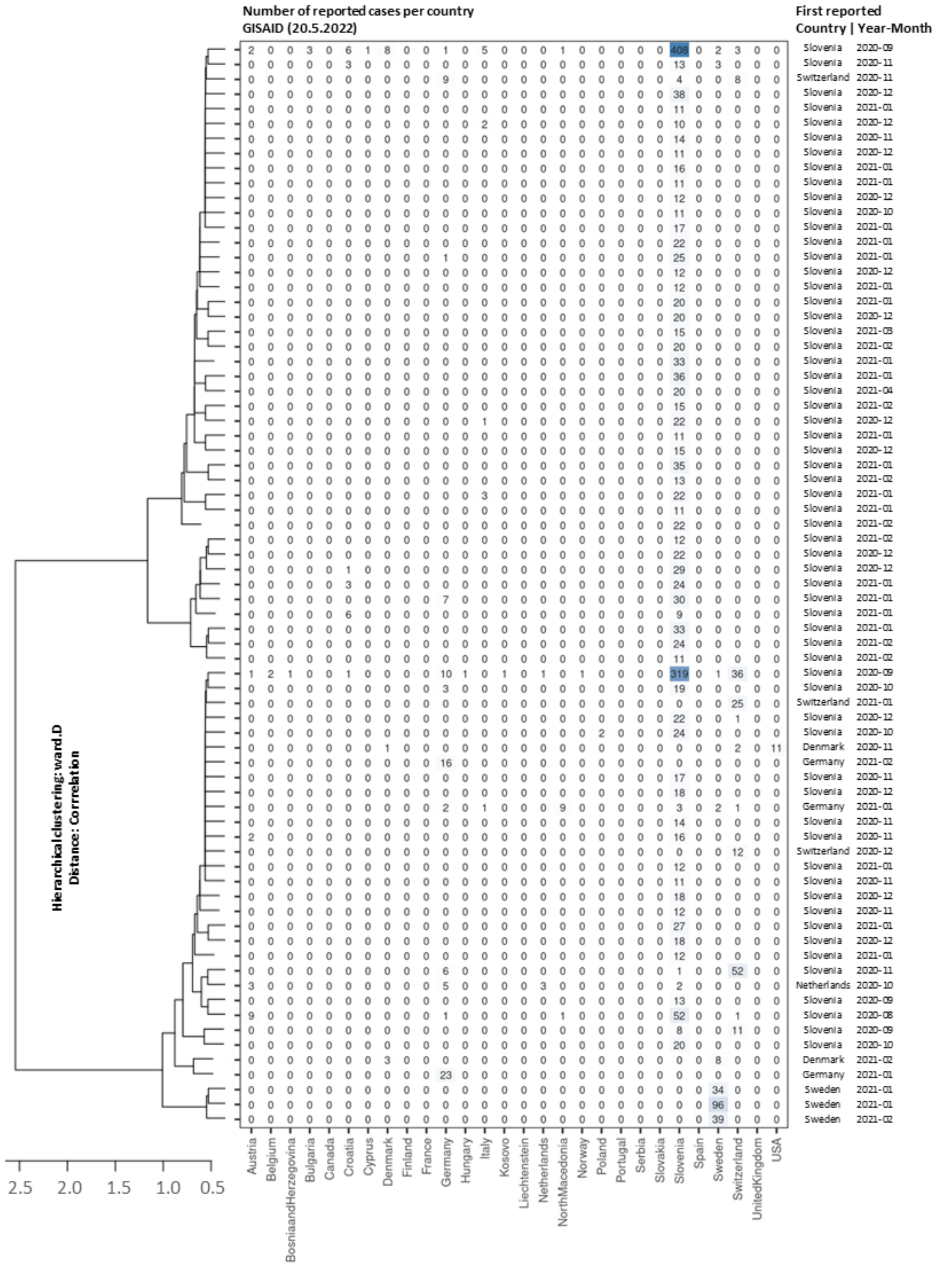

Supplementary Figure S3: Phylogenetic analysis of lineage B.1.258.17. Set of 4275 sequences belonging to B.1.258.17 lineage was obtained from GISAID (20.5.2022) implementing the following criteria: Lineage – B.1.258.17, Complete – yes, High coverage – yes. Based on amino-acid substitutions we made a seleciton of 73 most prevalent sub-lineages (n total >= 10 entries) and calculated philogenetic clustering using ‘ward.D’ clustering method and ‘correlation’ distances (R package ‘pvclust’). For each sub-lineage we present number of GISAID entries per country (heat-plot) and the information of the country and sample collection date (year-month) for the first uploaded instance of respected sub-lineage.

Supplementary methods

Library preparation, sequencing, and sequence analysis

Illumina compatible libraries were prepared with target genome amplification either with the ARTIC v3 protocol (URL: dx.doi.org/10.17504/protocols.io.bbmuik6w) or QIASEQ SARS-CoV-2 Primer Panel (QIAGEN). Both protocols were used according to published guidelines and recommendations or manufacturers manual. Partial library preparation followed by sequencing and bioinformatic analysis were performed at the Clinical Institute of Special Laboratory Diagnostics, University Children's Hospital, University Medical Center Ljubljana. Sequencing was performed on Illumina platforms, either MiSeq or NovaSeq 6000. The choice of kits and sequencing platforms depended on reagent availability. Sequencing was partially provided also by ECDC (n = 1,725 genomes).

Consensus genome sequences were obtained by 1) mapping reads to the reference genome (NC_045512) with BWA aligner (v 0.7.17) and 2) implementing the ivar (v 1.3)/samtools (mpileup, v 0.1.19) procedure for quality filtering, removing primer binding sites, and producing consensus sequences. SARS-CoV-2 variants were called with locally installed Pangolin (daily updated version; (3)), and mutations were obtained with online Nextclade analysis (4). Comparative genomics was performed by full genome alignment with mafft (v 7.480) and tree calculation with the IQ-TREE (v 1.6.1) GTR+G model. An exception was phylogenetic analysis of lineage B.1.258.17, for which a complete set of 6987 sequences belonging to the B.1.258.17 lineage was obtained from GISAID (20.5.2022) implementing the following criteria: Lineage – B.1.258.17, Complete – Yes, High coverage – Yes. Based on amino-acid substitutions, a selection of the 73 most prevalent sub-lineages was made (n total ≥ 10 entries), and phylogenetic clustering was performed using the ‘ward.D’ clustering method and ‘correlation’ distances (R package ‘pvclust’).

Virus isolation and growth kinetics using Vero E6 cells

Vero E6 cells were maintained in Eagle’s Minimum Essential Medium (EMEM, ATCC 30-2003) containing 10% FBS (Gibco) and 1% antibiotic-antimycotic (100X, Gibco) at 37 °C and 5% CO_2. Cells were routinely tested for mycoplasma and remained mycoplasma-free. All work with viruses in cell cultures was performed in a biosafety level 3 laboratory (BSL3) at the Institute of Microbiology and Parasitology, Veterinary Faculty, University of Ljubljana.

Samples of variants B.1.258.17 (n=6), B.1.258 (n=4), B.1.1.70 (n=3), Alpha/B.1.1.7 (n=3), and Delta/AY.43 sublineage (n=5) were included in the growth kinetics experiment. SARS-CoV-2 variants were isolated on Vero E6 cells from samples collected from SARS-CoV-2-positive individuals. Two or three passages of SARS-CoV-2 isolates were performed to prepare the working virus stock. Cytopathic effects were observed under an inverted microscope (Eclipse Ts2R, Nikon). The virus titers of the working stocks were determined by titration of the virus isolates on Vero E6 cells and measured as 50% infectious tissue culture

dose. To confirm the genomic sequences of the viral variants, RNA was extracted from the cell culture supernatant using the MagMAX™ CORE Nucleic Acid Purification Kit on the KingFisher Flex System (Thermo Scientific), and WGS was performed.

For the virus growth kinetics experiments, Vero E6 cells were seeded (3.5×10^5 cells/well) into 96-well microtiter plates and incubated at 37 °C and 5% CO_2. After 1 day, the cell monolayers were inoculated with 500 of 50% infectious tissue culture dose TCID50 of SARS-CoV-2 variants. Supernatants were collected at 2, 24, 72, and 120 h after inoculation. RNA was extracted from the supernatants using the MagMAX™ CORE Nucleic Acid Purification Kit on the KingFisher Flex System (Thermo Scientific). Each viral variant was tested in six replicates for each time point, and viral load from the supernatants was measured using the SARS-CoV-2 real-time PCR assay targeting the E gene (5).

All Cq values were normalized against measurements obtained after 2 h of incubation (considered as a baseline viral load), according to the equations (1) and (2) shown below.

(1) $\text{Weight}_{\text{(sample)}} = \text{Cq}_{\text{(sample at 2 h)}} / \text{Cq min}_{\text{(2 h)}}$;

where $\text{Cq}_{\text{(sample at 2 h)}}$ is the sample measurement at 2 h, and $\text{Cq min}_{\text{(2 h)}}$ is the lowest Cq measurement among all samples at the 2 h time-point.

(2) $\text{Normalized Cq}_{\text{(sample)}} = \text{Cq}_{\text{(sample)}} / \text{Weight}_{\text{(sample)}}$;

where $\text{Cq}_{\text{(sample)}}$ is the Cq measurement of the sample at consecutive time points.
